# Assessment of Body Composition in Athletes: A Narrative Review of Available Methods with Special Reference to Quantitative and Qualitative Bioimpedance Analysis

**DOI:** 10.3390/nu13051620

**Published:** 2021-05-12

**Authors:** Francesco Campa, Stefania Toselli, Massimiliano Mazzilli, Luís Alberto Gobbo, Giuseppe Coratella

**Affiliations:** 1 Department for Life Quality Studies, Università degli Studi di Bologna, 47921 Rimini, Italy; 2 Department of Biomedical and Neuromotor Sciences, Università degli Studi di Bologna, 40126 Bologna, Italy; stefania.toselli@unibo.it; 3 School of Sport and Exercise Sciences, Faculty of Psychology, “eCampus” University, 22060 Novedrate, Italy; massimilianomazzilli@gmail.com; 4 Department of Physical Education, São Paulo State University, Presidente Prudente 19060-900, SP, Brazil; luis.gobbo@unesp.br; 5 Department of Biomedical Sciences for Health, Università degli Studi di Milano, 20133 Milan, Italy; giuseppe.coratella@unimi.it

**Keywords:** bioelectric impedance vector analysis, BIVA, hydration, phase angle, localized BIA, nutritional status, segmental bioimpedance, tolerance ellipses

## Abstract

Body composition is acknowledged as a determinant of athletic health and performance. Its assessment is crucial in evaluating the efficiency of a diet or aspects related to the nutritional status of the athlete. Despite the methods traditionally used to assess body composition, bioelectric impedance analysis (BIA) and bioelectric impedance vector analysis (BIVA) have recently gained attention in sports, as well as in a research context. Only until recently have specific regression equations and reference tolerance ellipses for athletes become available, while specific recommendations for measurement procedures still remain scarce. Therefore, the present narrative review summarizes the current literature regarding body composition analysis, with a special focus on BIA and BIVA. The use of specific technologies and sampling frequencies is described, and recommendations for the assessment of body composition in athletes are provided. Additionally, the estimation of body composition parameters (i.e., quantitative analysis) and the interpretation of the raw bioelectrical data (i.e., qualitative analysis) are examined, highlighting the innovations now available in athletes. Lastly, it should be noted that, up until 2020, the use of BIA and BIVA in athletes failed to provide accurate results due to unspecific equations and references; however, new perspectives are now unfolding for researchers and practitioners. In light of this, BIA and especially BIVA can be utilized to monitor the nutritional status and the seasonal changes in body composition in athletes, as well as provide accurate within- and between-athlete comparisons.

## 1. Introduction

Body composition and nutritional status are acknowledged as determinants of athletic health and performance [1]. Indeed, in many sports, an athlete can gain an advantage by changing their body mass or body composition features. For example, sports such as gymnastics include both an aesthetic and a gravitational component; thus, anthropometric characteristics may affect a gymnast’s success in competitions [2]. Moreover, many sports are weight-classified; therefore, athletes must stay within a certain body mass range [3]. Consequently, athletes carefully adjust their training and nutritional habits depending on the specific sports demands [4]. In this context, monitoring body composition has become crucial, and assessing it appropriately allows for an accurate evaluation. 

Body composition describes and quantifies various elements within the human body [5]. It was pioneeristically proposed that body composition can be approached on the basis of five levels of increasing complexity, considering body mass as the sum of atoms, molecules, cells, tissues, or different body segments [6]. In each model, a series of components from atoms to body segments qualitatively describe body mass [7,8,9]. The appropriate approach to assess body composition should consider the parameters derived from each level separately, so that the sum of each within-level parameter determines the body mass. As such, (i) the atomic level considers the amount of hydrogen, carbon, oxygen, and other atoms [7,8,9], (ii) the molecular level encompasses the fat mass and fat-free mass that embed total body water and bone mineral content [7,8,9], (iii) the cellular level includes adipose cells, intracellular and extracellular water, and body cell mass [7,8,9], (iv) the tissue level examines the amount of adipose and lean soft tissue and skeletal muscle mass [7,8,9], and (v) the whole body level sums up the mass of different body segments (i.e., head, trunk, and limbs).

**Figure 1:**
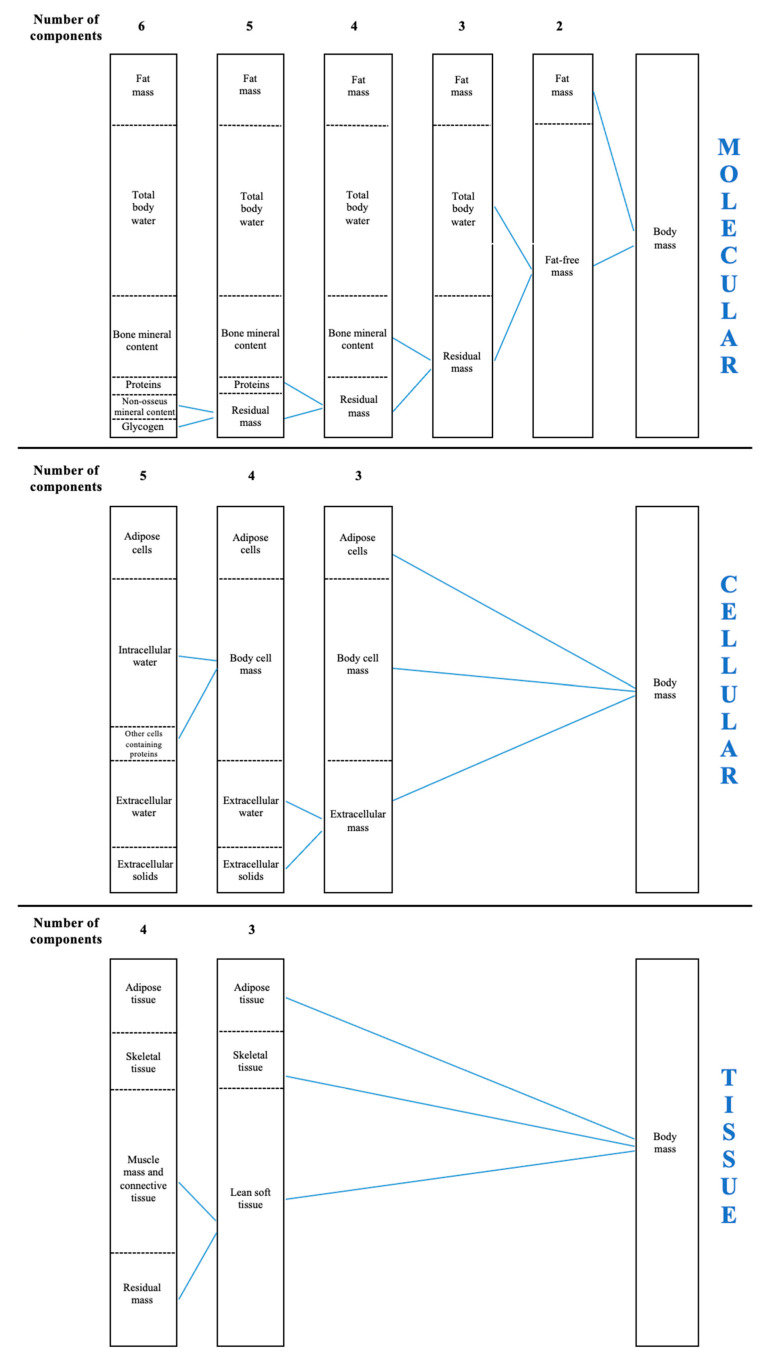
The compartment models to evaluate body composition are shown.

On the basis of the five levels, several models have been implemented to assess body composition (Figure 1). In clinical and research contexts, the four-compartment molecular model and the three-compartment tissue model are largely used to assess body composition [10]. However, when assessing body composition in the sports field, some parameters derived from different models are independently considered, for example, fat mass from the molecular level, body cell mass and intra/extra cellular water from the cellular level, or muscle mass from the tissue level. Although this might provide some practical information, combining parameters from different levels does not allow for the accurate assessment of body composition, as each parameter should be evaluated separately. Therefore, practitioners aiming to assess body composition should consider the parameters derived from one of the aforementioned methods, to avoid possible misinterpretations. 

Once the model has been chosen, each parameter should be assessed using its reference method, to achieve the greatest accuracy. These methods can be classified into direct, indirect, or double-indirect approaches [11,12,13,14], as shown in Table 1. Direct methods measure a given parameter directly, while indirect methods use assumptions or algorithms to estimate it. Lastly, double-indirect methods use validated regression equations, with estimations derived from indirect methods. However, indirect methods can still be considered as reference methods or gold standard when a specific body composition parameter is difficult to obtain in vivo or to measure on a large scale [15]. At the molecular level, dual-energy X-ray absorptiometry (DXA) is considered the reference method to determine bone mineral content [7,9], while the deuterium dilution technique is the reference procedure for assessing total body water [7]. Additionally, hydrostatic weighing and air plethysmography are considered the gold standard for assessing fat mass [7]. However, as concerns fat mass in the four-compartment model, a specific formula is considered the most accurate approach, and it requires the assessment of bone mineral content by DXA, total body water by deuterium dilution, and body volume by air displacement plethysmography [8]. As such, the fat mass can be calculated as follows: fat mass (kg) = 2.748 × body volume − 0.699 × total body water + 1.129 × bone mineral content − 2.051 × body mass [10]. At the cellular level, body cell mass is measured using the whole-body counting procedure, while the bromide dilution allows for the estimation of extracellular water. At the tissue level, imaging techniques such as DXA and magnetic resonance are considered the reference methods to determine lean soft tissue and muscle mass. Hence, some considerations can be made: firstly, indirect methods are largely considered as gold standard to assess a given parameter; secondly, it is clear that a single device or technique cannot be identified as a gold standard for assessing whole-body composition but should refer to the warranted parameters; lastly, no in vivo technique may be considered to meet the highest criteria of accuracy in the body composition analysis.

Notwithstanding, most methods and/or devices are expensive, are time-consuming, and require qualified personnel, limiting both the research and the sports contexts. As a response to overcome such issues, bioelectric impedance measurement was introduced in the 1960s [16] and then further implemented in the 1980s [17]. To date, bioelectrical impedance spectroscopy (BIS) and bioelectric impedance analysis (BIA) represent two double-indirect methods based on the assessment of body impedance [17]. The BIS method was the first proposed approach for measuring impedance and uses a range of frequencies (4 to 1000 kHz) to estimate impedance and phase angle, from which bioelectrical reactance (Xc) is calculated. The derived parameters are then used in nonlinear mathematical models to estimate intra- and extracellular resistance values [18]. In contrast, when using phase-sensitive devices, the impedance can be separated into bioelectrical resistance (R) and Xc, and the terms single- and multi-frequency BIA are used for this type of analysis [19,20]. Single-frequency BIA refers to the measurement technology that performs measurements at a single frequency. On the contrary, multi-frequency BIA applies a technique employing data collection at more than one frequency. Lastly, in order to clearly distinguish multi-frequency BIA from the analysis based on Cole plots or other models for fitting impedance data over the entire frequency range (between 1 kHz and 1000 kHz), the term BIS has also been frequently used to refer to the latter [20]. Particularly, the impedance includes the resistance (R), the force that a biological conductor opposes to an alternating current attributable to intracellular and extracellular fluids, and the reactance (Xc), arising from the cell membranes and representing the capacitive component of the impedance [21,22]. This allows fluids and their distribution to be determined. Starting from the unique impedance properties of each tissue, several regression equations have been implemented to obtain a number of body composition parameters [20,23]. Current application of BIS and BIA aims to predict body composition parameters for inclusion in multicomponent models. However, only single-frequency BIA has been used to develop prediction equations to estimate different body composition parameters in athletes [24,25,26].

Since BIA is cost-effective, portable, and time-efficient, its use in both research and sports practice has rapidly increased in recent years [23,27,28]. However, some concerns have been raised when using BIA. First, several devices with different technologies have been designed and are currently used; thus, an inter-device comparison cannot be made. Indeed, each device with its own technology outputs a range of values, depending on the sampling frequency and the device’s reliability [23,27,28]. Second, the procedures should be standardized, since different electrode placement, calibration, body position, skin preparation, nutritional status, circadian rhythm, and acute training status may affect the results. Third, while BIA was initially designed for the general population, assessing body composition using unspecific regression equation results in inaccurate findings when assessing athletes. Lastly, some devices provide raw data to be inserted into regression equations, while others output the body composition parameters, limiting the possibility of a further qualitative analysis [23,27,28]. 

## 2. Aim of Narrative Review

Since 2003, several articles have been published using BIA to assess the body composition in athletes. However, given the very specific physical features of athletes, as well as the sport-specific secular trend [29], the use of tailored regression equations and references is warranted [23,28]. For this reason, a previous review called for action by the scientific community, since such regression equations were not available [23]. Most recently, some regression equations have been proposed for athletes, distinguishing the male and female population [24,25,26]. Similarly, new elements for a qualitative analysis in sport-specific populations have been developed [30,31,32,33,34,35,36]. Therefore, the present narrative review aimed to summarize the current literature regarding BIA, emphasizing the characteristics of the different technical approaches, as well as their limitations. Moreover, possible novel applications and future recommendations and perspectives are discussed.

## 3. Methods

A literature search was performed to identify relevant articles to include in this narrative review; a description of the search strategy and screening process is provided in Appendix A and Supplementary Table S1. Studies were considered relevant if they recruited an athletic population; this includes individuals competing in any individual or team sport that demonstrate a high level of conditioning or train at least four times per week. To be considered relevant, the articles must also have measured and evaluated bioelectric parameters (e.g., R, Xc, phase angle, and vector length) or estimated body composition using predictive bioimpedance-based equations. 

## 4. Bioelectric Impedance Analysis (BIA) in Athletes

**Figure 2:**
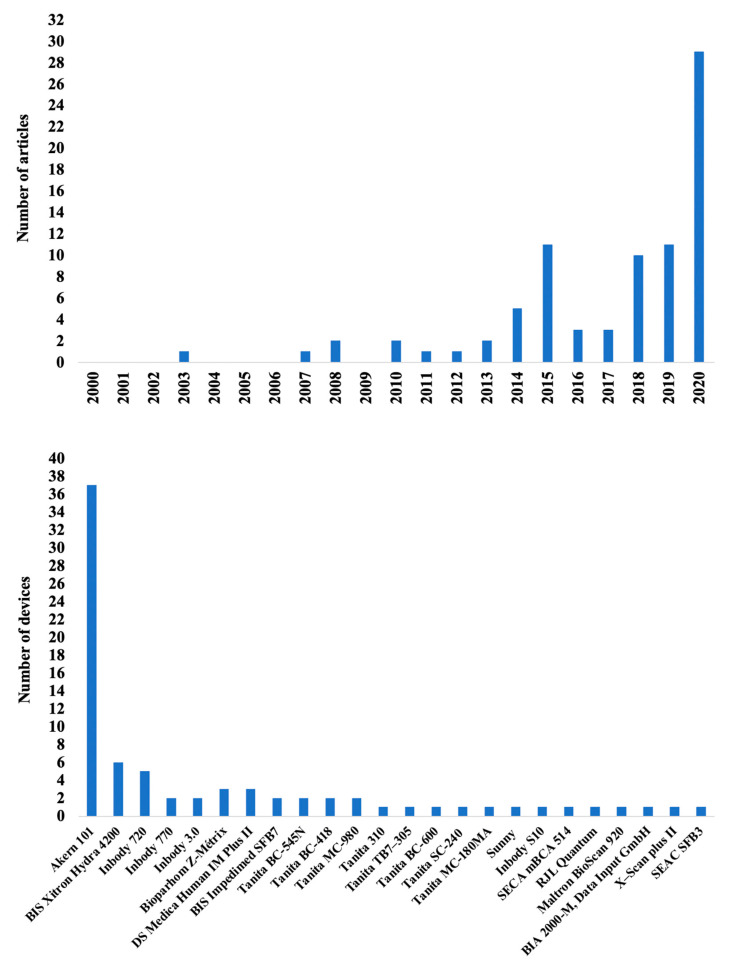
The number of articles per year from 2000 to 2020 using bioimpedance in athletes (upper panel), and the number of articles per device (lower panel) are shown.

The annual rate of papers publishes listing BIA in assessing body composition in athletes has increased rapidly since 2000 (Figure 2). A number of commercial BIA devices were used in these articles (number of devices = 24) (Figure 2).

From 2000 to 2014, BIA was used in 15 studies [34,35,37,38,39,40,41,42,43,44,45,46,47,48,49], with a peak of 11 articles published in 2015 [50,51,52,53,54,55,56,57,58,59,60], followed by a decline 2 years later [24,61,62,63,64], before undergoing a progressive increase beginning in 2018 up until 2020 [65,66,67,68,69,70,71,72,73,74,75,76,77,78,79,80,81,82,83,84,85,86,87,88,89,90,91,92,93,94,95,96,97,98,99,100,101,102,103,104,105,106,107,108]. Possibly, all articles published before 2018 mainly used the quantitative assessment of body composition, i.e., the simple estimation of the different body composition parameters using prediction equations. However, no specific formulas developed and validated for athletes were available at that time; thus, the equations used in these studies were those proposed for the general population. This may have led to inaccurate values, generating doubts about the accuracy and usefulness of BIA in athletes [23,27,28]. These perplexities have pushed researchers to develop specific equations [24,25,26] or use alternative evaluation approaches [31,45,56,78,109,110].

The first alternative approach came in 2015, when the segmental BIA was used to estimate the body composition of different body segments in athletes for the first time [51]. Indeed, the segmental BIA allows for the independent assessment of the individual body segments, defined as the four limbs and the trunk [110]. Alternatively, in 2013, the evaluation of the bioelectrical properties in specific body segments in soccer players was proposed, an approach coined localized BIA (L-BIA) [45]. The L-BIA was thought to evaluate the recovery status after strain injuries, measuring the changes in R and Xc to assess the changes in fluids and cell integrity, respectively [45]. It is critical to highlight how both segmental and L-BIA measurements are regional analyses but involve different procedures. In the measurement of segmental bioimpedance, the two injector electrodes are located and fixed in the metacarpophalangeal and metatarsophalangeal joints, while the sensing electrodes are usually placed at the end of the superior and inferior limbs [111,112,113,114,115,116]. On the contrary, in L-BIA measurements, the four electrodes are applied on the body region of interest, such as specific muscles [45,58,93,117,118].

Alternatively, the bioelectrical phase angle represents a qualitative approach to the body composition analysis assessed by BIA and is calculated as the arctangent of Xc/R × 180°/π [22]. Graphically, it is represented as the angle between the vector and the *x*-axis [20], and it is considered a nutritional status index [41]. As a further alternative, bioelectric impedance vector analysis (BIVA), initially proposed by Piccoli et al. [119] in 1994, was used for the first time in 2007 [46]. BIVA consists of the simultaneous evaluation of the raw parameters recorded in BIA (i.e., R and Xc), plotting them as a vector within a graph [119]. BIVA identifies the changes in body fluids and hydration status [18,120], and its accuracy was confirmed upon comparing BIVA to the dilution technique as reference method [70,88,102]. To date, a number of studies have compared the results coming from BIA or BIVA with the reference methods, as reported in Table 2.

## 5. BIA Evaluation Procedures

**Figure 3:**
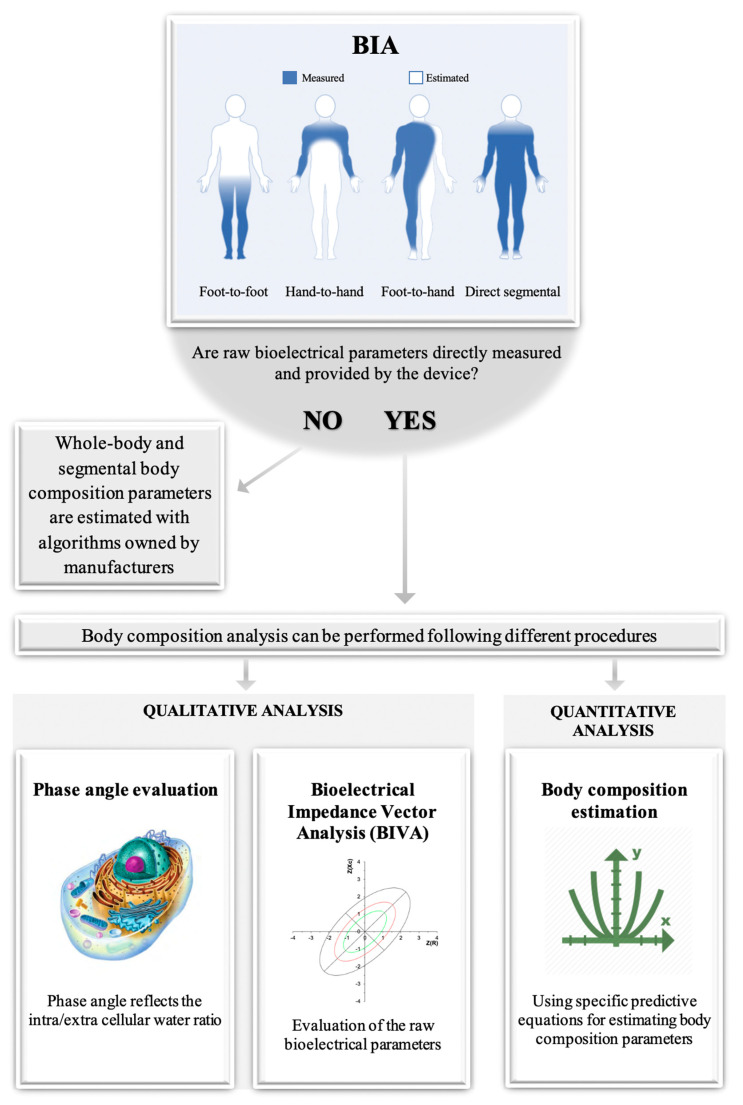
The paradigm of the bioelectric impedance analysis is shown.

Between-device and within-device differences in the BIA-derived parameters can be obtained when varying the technologies and procedures, respectively. The between-device differences depend on four different technologies: hand-to-hand, foot-to-foot, direct segmental, and foot-to-hand [121,122,123,124]. The hand-to-hand technology measures the upper body impedance, while estimating the rest of the body through dedicated algorithms. On the contrary, the foot-to-foot technology measures the lower body impedance, while estimating the rest. In contrast, the direct segmental technology measures the whole-body impedance. These three technologies are between-operator consistent, since the body composition outcomes are directly output from the device, and the procedures do not depend on the operator’s experience. It should be noted that some direct segmental devices provide raw BIA data (e.g., Inbody 720); hence, the operator must insert them into specific regression equations to get body composition outcomes. Indeed, while the previous technologies use a scale platform and/or handgrip electrodes, the foot-to-hand is based on the impedance recorded by four or eight electrodes for the estimation of whole-body or segmental body composition parameters, respectively [121,122,123,124]. As such, the electrode placement affects the raw data output, thus possibly showing between-operator differences [123,124,125]. Hence, the raw data could be used for a quantitative or qualitative approach in the assessment of body composition. A brief summary is depicted in Figure 3.

Irrespective of the technology used, both between- and within-device differences in output data may exist depending on the sampling frequency. Indeed, a previous study reported differences in raw bioimpedance parameters when different sampling frequencies were used [101]. Several devices have sampling frequencies ranging from 5 kHz to 500 kHz, although some of them may reach 1000 kHz [121]. Low frequency (e.g., 5 kHz) can only provide information on the extracellular water, since the cell membrane cannot be penetrated [20]. On the contrary, at high frequency, the current can flow through both extra- and intracellular compartments [20]. However, poor reproducibility has been observed at frequencies below 5 kHz and above 200 kHz [121]. To overcome such issues, an intermediate frequency of 50 kHz was proposed as the best sampling frequency [123,124]. It should be noted that some devices allow multi-frequency sampling to be recorded over a range of frequencies, i.e., bioelectrical impedance spectroscopy [20]. Typically, spectroscopy devices use Cole modeling and mixture theories rather than regression equations to assess body composition parameters [20]. Notwithstanding, the number of frequencies needed before a BIA device can be considered a spectroscopy device is not clear [20].

**Figure 4:**
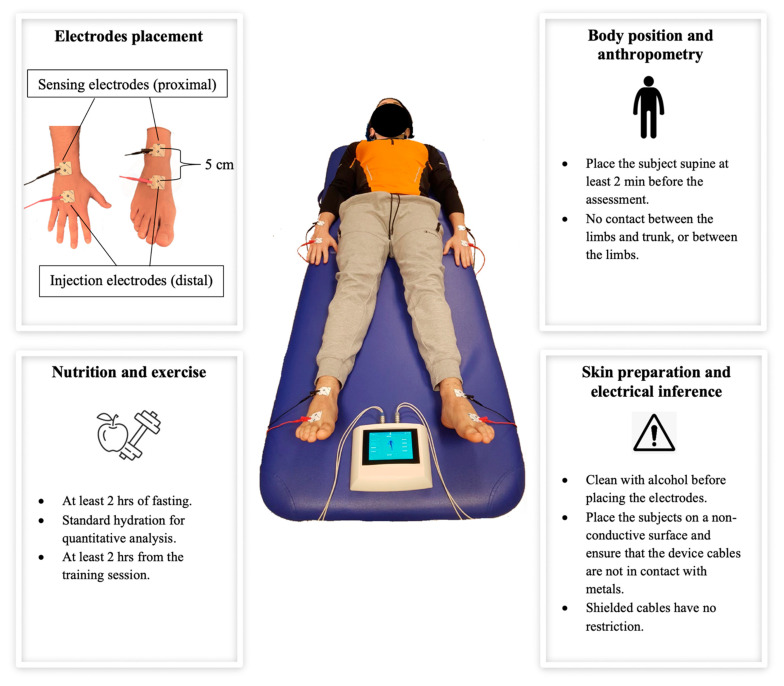
The recommendations for the measurement procedures using bioimpedance analysis are depicted and summarized.

To date, the foot-to-hand technology at 50 kHz single frequency is considered the reference method for BIA in humans [123,124]. As also mentioned above, the foot-to-hand technology consists of placing four or eight electrodes on the body, such that each electrode’s placement may affect the output, and between-operator differences may occur. Additionally, other possible confounding factors are the body position, previous exercise, and diet [123,124]. All these independent parameters should be standardized, so that valid and reliable procedures can be used. Nevertheless, most of the studies failed to report how the procedures were conducted, making a between-study comparison challenging. The procedure recommendations for the clinical applications of BIA in the general and pediatric population were previously reported [123,124,125]. However, such recommendations were not implemented for athletes. Therefore, general recommendations for BIA using foot-to-hand technique in athletes are shown in Figure 4 and integrated below.

- Device: the frequency, the amperage, and the type of signal measured (i.e., impedance or R or Xc or phase angle) should be reported.
- Electrodes: the type and size of the electrodes supplied by the manufacturer should be reported. The recommended electrode placement is depicted in Figure 4.
- Calibration: an electronic verification module with a tolerance of ±1% to assess the accuracy of the device should be used.
- Anthropometry: the body mass and stature should be reported to the nearest ±0.1 kg and ±0.5 cm, respectively, and measured each time.
- Environment: the measurement should occur at an external temperature ranging from 22.3 °C to 27.7 °C (72.1 °F to 81.9 °F).
- Time of measurement: cross-sectional between-subject assessment should be performed within the same competitive period for each athlete. Circadian rhythms should be taken into account; thus, longitudinal within-subject measurements should be performed at the same time of the day.
- Menstrual cycle: the phase of the menstrual cycle should be specified, and both within- and between-subject body composition assessments in women should be performed in the same phase of the menstrual cycle.
- Body composition assessment: validated regression equations and BIVA tolerance ellipses for athletes should be used. Additionally, both regression equations and BIVA tolerance ellipses should refer to the frequency used to validate them.

## 6. Quantitative Analysis: Estimation of Body Composition Variables through Predictive Equations

The main use of BIA in athletes involves the estimation of body composition absolute (kg or L) or relative (%) parameters through predictive equations. This is possible thanks to the conductance properties of each biological tissue. More in detail, the highly hydrated fat-free mass is a good electrical conductor, while the poorly hydrated adipose tissue is an electrical insulator [22]. Therefore, the total body water and the impedance are negatively correlated, and the changes in the former also affect the changes in the latter. Additionally, Lukaski et al. [17] introduced the impedance index, defined as the stature (cm)^2^/R (ohm) ratio, which is based on Ohm’s law that states that a volume of constant section is proportional to the length squared divided by its resistance. Such an impedance index was shown as predictive of the fat-free mass, total body water, and body cell mass [17]; it was, thus, inserted into all regression equations for the estimation of the body composition. 

Bioelectric impedance-based regression equations, which typically include stature, weight, age, and sex, transform the measured electrical impedance and its components of R and Xc into volume (intracellular, extracellular), mass (fat mass, fat-free mass, body cell mass), and other variables. In general, these estimators are more susceptible to violating body composition assumptions, especially those regarding tissue hydration [9,119,126,127,128]. In such cases, total body water is estimated using R, and fat-free mass is estimated from the former by assuming a constant soft-tissue hydration, while fat mass is subsequently calculated as the difference between body weight and fat-free mass [9,126,127,129]. Additionally, in most of these predictive equations, the Xc component is not included in the predictive models.

To date, a number of regression equations have been implemented for athletes, as reported in Table 3. Such equations showed high predictive capacity and low error for each parameter (total body water: *R^2^* = 0.93, SEE = 2.42 kg; extracellular water: *R^2^* = 0.84, SEE = 1.33 kg; fat-free mass: *R^2^* = 0.94, SEE = 3.0 kg; arm lean soft tissue: *R^2^* = 0.89, SEE = 0.62 kg; leg lean soft tissue: *R^2^* = 0.81, SEE = 1.95 kg), and they require only body mass and stature, in addition to R and Xc, to be inserted [24,25,26]. These equations were developed for both sexes and subsequently validated on different groups of athletes [24,25,26].

**Figure 5:**
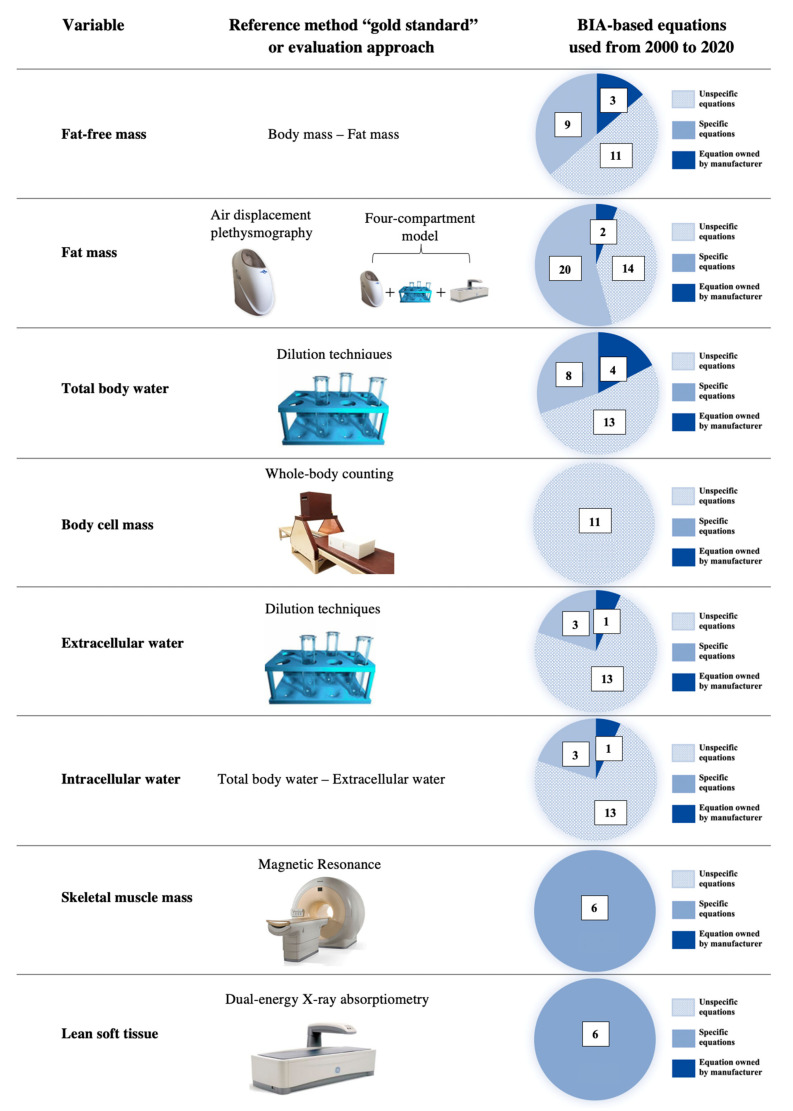
The body composition parameters assessed by the bioimpedance analysis in the literature are listed (**left column**). The reference method for assessing each parameter is shown in the central column. The number of studies using unspecific, specific, or manufacturer regression equations is shown (**right column**).

Figure 5 shows the body composition parameters, their reference methods, and the number of articles that used specific, unspecific, or manufacturer regression equations for assessing body composition in athletes. To date, few studies have used specific regression equations, while most of the literature refers to unspecific or unknown regression equations. This may be due to the very recent availability of specific formulas to predict total body water and extracellular water (starting from 2016) or fat-free mass and lean soft tissue (starting from 2020). More importantly, body cell mass and skeletal muscle mass still do not present any specific regression equation validated for athletes. This may depend on the complex procedures required when using the reference method for assessing the body cell mass (i.e., whole-body counting) or long duration when using the reference method for assessing the skeletal muscle mass (i.e., magnetic resonance). Remarkably, BIA can be used to assess a wide range of body composition parameters, which theoretically require a dedicated device to be easily used in practice.

## 7. Qualitative Analysis: Interpretation of the Raw Bioimpedance Parameters

### 7.1. Bioelectrical Phase Angle and Localized Bioimpedance Analysis (L-BIA)

The evaluation of the phase angle is a qualitative approach included in the analysis of the body composition through BIA [22]. The bioelectrical phase angle represents a qualitative approach to the body composition analysis assessed by BIA and is calculated as the arctangent of Xc/R × 180°/π (Lukaski and Piccoli 2012). Graphically, it is represented as the angle between impedance and the *x*-axis (Stahn et al. 2012). Previous studies, using dilution techniques as reference, have shown how phase angle mainly represents the intra/extracellular water ratio [70,88], whose changes may indicate fluid shifts between the compartments, as a result of cell damage, inflammation, or dehydration [45,64,93,98,120]. As such, the phase angle has been proposed to assess body composition using whole-body, segmental, or L-BIA. Indeed, higher phase angle values are associated with higher muscle mass or acute dehydration, while lower phase angle values are related to lower muscle mass, acute hyperhydration, or chronic dehydration [31,46,98]. However, although higher phase angle values can be found in elite vs. sub-elite athletes participating in the same sport [32,33,34], the ability to discriminate athletes from different sports is debated [130]. As such, the phase angle should be used to monitor the within-athlete changes in body composition over time but should not be used for a between-athlete comparison. Lastly, phase angle is used in L-BIA to check for the recovery of muscular strain injury, where lower values indicate an inflammatory status due to the increment of extracellular fluid after a cellular rupture [45,58].

### 7.2. Bioelectrical Impedance Vector Analysis (BIVA)

**Figure 6:**
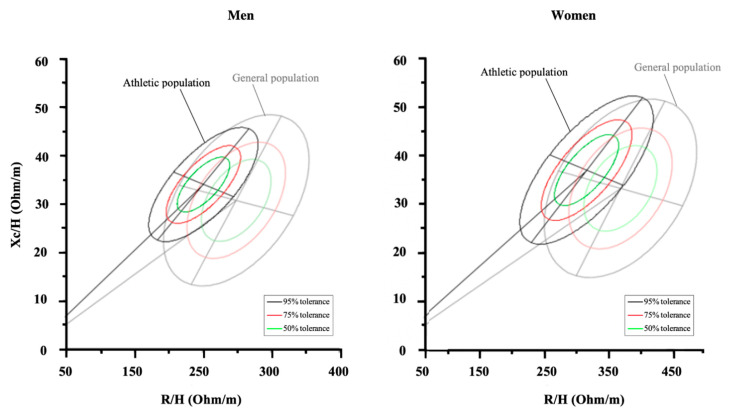
The reference tolerance ellipses for general and athletic populations are shown.

The qualitative analysis through BIVA consists of the interpretation of the raw bioimpedance parameters and avoids the typical concerns associated with the use of regression equations. BIVA’s ability to properly assess body composition over time was compared with gold-standard methods, such as the four-compartment model [88], dilution technique [102], and DXA [70], showing interchangeable results when assessing body fluid and soft-tissue changes in athletes during the competitive season. It is not possible to estimate body composition parameters (e.g., fat mass, fat-free mass, total body water) using BIVA, but the vector position can be evaluated within tolerance ellipses drawn for each specific population [31,119,131]. Such tolerance ellipses reflect the percentile in body composition parameters and may help to identify the specific athlete’s profile for each sport [31]. The first athlete-specific tolerance ellipses were provided to soccer players in 2014 [34]. In fact, the use of BIVA allowed for the possibility to present reference target zones, not only for a specific sport but for each competitive level. On the basis of these findings, numerous studies then provided specific tolerance ellipses for each sport and for different categories using BIVA (Table 4), even specific ellipses based on the competitive period [30]. Interestingly, BIVA patterns were shown to be able to discriminate elite from sub-elite athletes within the R-Xc graph, as reported in cycling [33], soccer [34], and volleyball [32] athletes. Moreover, BIVA was able to discriminate power/velocity, team sports, or endurance athletes in both sexes [31]. Additionally, BIVA was used to monitor the weight cut strategies in boxers [47] and judo athletes [102]. This latter study also showed the ability of BIVA to replicate the changes in body fluids as assessed by the dilution techniques as a reference method [102]. Furthermore, weekly fluctuations in BIVA vector were described to reflect the recovery time-course or the training-induced adaptations [100]. Intriguingly, BIVA was also used to evaluate the maturity status in adolescent soccer players, extrapolating possible differences in maturity status [36,72,86]. Figure 6 shows how the athletic population has different bioelectrical properties compared to the normal population. While the ellipses of the athletic population [31] are more shifted to the left than the general population [131], some sports categories such as cyclists show a vertical upward position [31,33]. Therefore, athletes must be considered in appropriate tolerance ellipses, which are currently available for different sports (Table 4). 

**Figure 7:**
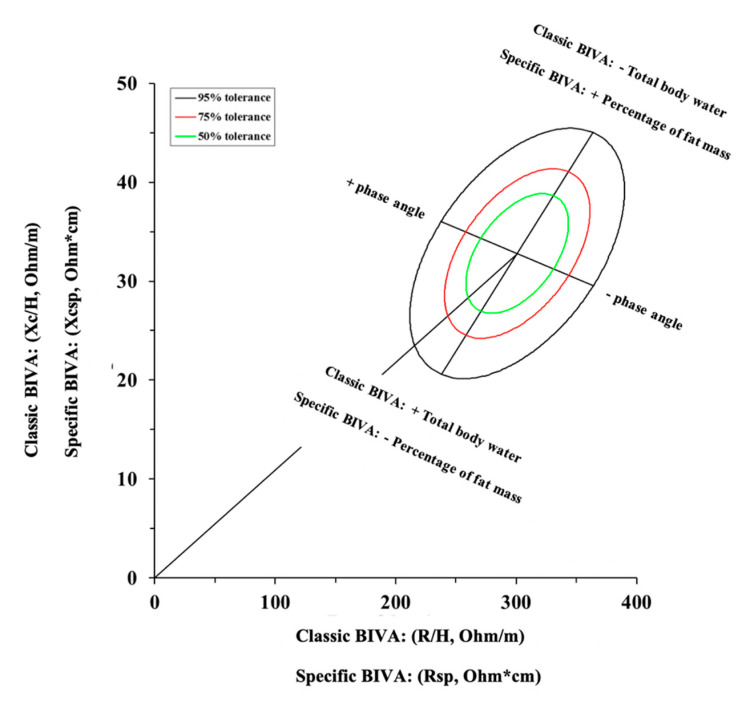
Classic and specific Bioelectrical Impedance Vector Analysis (BIVA).

As an alternative, in 2013, a variation in the classic BIVA was proposed [109], and it was recently used to assess body composition in athletes [36,88]. According to this alternative approach named “specific BIVA”, R and Xc are adjusted concurrently for the cross-sectional area of the arm, waist, and calf [109]. Specifically, the cross-sectional area of each body segment can be estimated as follows: segment area = circumference^2^/4π, where circumference is expressed in meters and refers separately to arm, waist, and calf. Thereafter, the following equation is used to adjust R and Xc into the specific BIVA: area = (0.45(arm area) + 0.45(calf area) + 0.10(waist area) (m^2^)). As such, the classic BIVA assesses the changes in body fluids, while the specific BIVA assesses the changes in percentage of fat mass [88,109], as shown in Figure 7. 

## 8. Quantitative and Qualitative Analysis for Assessing Hydration and Nutritional Status 

Body composition is determined by the quantity and quality of several elements, impacting performance and health in athletes. Weight cutting is a popular strategy adopted in some sports, and monitoring the hydration status and the body fluid distribution is crucial in this context [47,132]. Furthermore, maintaining an optimal fluid balance is essential in order to preserve physical and mental performance and, therefore, the evaluation of body fluids is necessary when facing close competitions. Through quantitative analysis, it is possible to estimate and evaluate the total body, intracellular, and extracellular water content in relation to body mass or fat-free mass; on the other hand, using qualitative analysis, it is possible to monitor the vector position within the R–Xc graph. Evaluating athletes using appropriate population references enables BIVA to classify (i.e., normal, under, and over) and rank (i.e., change relative to pretreatment) hydration, regardless of the body mass [18]. Similarly, it is difficult to obtain direct information about the state of hydration with a single BIVA, while it is possible with at least two measurements performed in a short period of time [97,98,107,120]. In particular, vector stretches after a sport performance identify reductions in total body water and, therefore, dehydration [97,98,120]. Furthermore, the assessment of the body fluid distribution can also be performed considering the phase angle, as mentioned in the previous paragraphs. In addition to the relationship with the intracellular/extracellular water ratio, phase angle has been reported to be positively associated with most nutritional markers and is an indicator of membrane integrity [76]. In this regard, phase angle monitoring may provide useful information about the effects of supplementation strategies during a training program. Although not concerning athletes, some studies examined phase angle changes in response to different supplementation strategies. For example, isocaloric dietary regimes with a protein content of 1.8 g.kg^−1^.day^−1^ or 2.9 g.kg^−1^.day^−1^ have been shown to affect phase angle differently during a 10 day resistance training program, in which phase angle increments were measured only after the higher protein supplementation period [133]. Furthermore, a recent study highlighted that, when consuming a high-protein diet, none of the α-hydroxyisocaproic acid, β- hydroxy-β-methylbutyrate free acid, and calcium β-hydroxy-β-methylbutyrate metabolites induce changes in phase angle in resistance-trained men, suggesting that supplementation with leucine metabolites is not a supplementation strategy that improves cellular integrity and induces ergogenic effects during a resistance training program [134]. 

## 9. Conclusions

The use of BIA and BIVA to assess body composition in athletes has been gaining popularity. The fact that BIA is a noninvasive, quick, relatively low-cost, and technologically simple method using portable equipment makes it easily usable in both research and practical application. Moreover, BIA allows for the estimation of a wide range of body composition parameters, following a whole-body or segmental approach. Although the regression equations for athletes were unavailable for a long time, a number of regression equations dedicated to the athletic population have now been developed. This permits a more accurate quantitative analysis of the body composition than using unspecific formulas. However, some devices do not allow the use of specific equations, since no raw data are provided. In addition to this quantitative analysis, qualitative analysis can also be used to monitor changes in bioelectrical parameters and, hence, in body composition, by comparing them with population-specific BIVA references. In this regard, athlete-specific tolerance ellipses are also now available. Similar to quantitative analysis, it should be mentioned that only devices which provide raw data allow the use of qualitative analysis. Future studies should try to provide specific regression equations for estimating body cell mass and skeletal muscle mass. 

In practice, some recommendations and practical applications should be highlighted. The use of BIA should always refer to a consistent environment and location where the assessment is performed. Indeed, changes in both temperature and humidity may result in artefacts. For similar reasons, the assessment should take place after a consistent time lag from the training session or competition. Remarkably, both quantitative (e.g., fat mass and body fluids) and qualitative (e.g., vector position and phase angle) parameters can be used as markers to address specific training cycles, depending on the period of the competitive season. However, qualitative analysis does not provide accurate detection of the hydration status from a single measurement; thus, multiple assessments should be performed over time. Notwithstanding, the appropriate use of BIVA may highlight fluid loss over time, especially useful where weight cutting is required. Lastly, the evaluation of phase angle for assessing the effect of supplement strategies on cellular integrity and nutritional status represents an interesting topic for future research on sports nutrition.

## Figures and Tables

**Table 1:** Comparative advantages and disadvantages of a selection of in-vivo body composition assessment methods.

	Method	Advantages	Disadvantages
**Direct**	Whole body counting	High accuracy	Costs, technical difficulties.
**Indirect**	Densitometry (underwater weighing, air-displacement plethysmography)	Relatively fast and non-invasive	Costs, hydration assumptions, effects of disease on lean mass reduce accuracy, distribution of fat unable to be determined.
	Hydrometry (D_2_O, NaBr)	Suitable for all age group	Costs, low acceptability, delayed results.
	Dual-energy X-ray Absorptiometry (DXA)	Reliable and repeatable. Can provide regional as well as total evaluations	Small radiation exposure. Can overestimate fat mass.
	Magnetic resonance, computed tomography	High reproducibility, accurate assessment of lean soft tissue, assessment of regional adiposity and of intra-abdominal vs subcutaneous adiposity.	Costs, not suitable for all infants due to need for transfer to scanner and time required for scan acquisition. Computed tomography involves the use of X-rays, which are a form of ionizing radiation.
**Double Indirect**	Anthropometry	Simple measurement of subcutaneous fat	Population specific, poor accuracy in individuals and groups, training required.
	Bioelectric Impedance Analysis (BIA)	Quick and non-invasive. Cumulative accuracy makes useful for repeated measures	Population specific. Distribution of fat unable to be determined.

**Table 2:** Studies comparing bioimpedance outcomes and bioimpedance-derived body composition parameters with reference methods in athletes.

Authors	Aim	Study Design	Participants	Technology and Sampling Frequency	Reference Method	Results
Esco et al. (2015) [51]	Assessing the agreement between multifrequency BIA and DXA for measuring fat mass, fat-free mass, and total body and segmental lean soft tissue	Cross-sectional	45 female athletes (age 21.2 ± 2.0 year) engaged in different sports	Direct segmental at multifrequency	DXA	(i) Multifrequency BIA underestimated fat mass and overestimated fat-free mass (ii) Multifrequency BIA and DXA showed agreement for measuring total body and segmental lean soft tissue
Raymond et al. (2018) [108]	Assessing the agreement between multifrequency BIA and DXA for measuring fat mass and fat-free mass	Cross-sectional	44 male athletes (age 19.6 ± 1.0 year) collegiate football athletes	Direct segmental at multifrequency	DXA	(i) Multifrequency BIA underestimated fat mass and overestimated fat-free mass
Domingos et al. (2019) [79]	Assessing the validity of BIA to determine fat mass and fat-free mass	Cross-sectional study	29 male judo athletes (age 23.1 ± 3.4 year)	Foot-to-foot at multifrequency	Four-compartment model	(i) BIA overestimated fat mass, while showed agreement for measuring fat-free mass
Silva et al. (2019) [102]	Assessing the ability of BIVA in tracking body fluids changes during the preparation period prior to competition in combat sport	Observational study	27 male judo athletes (age 23.2 ± 2.8 year)	Foot-to hand at 50 kHz	Dilution techniques (deuterium and bromide)	(i) Decreases in total body water were accompanied by vector elongations, and vice versa (ii) Changes in intracellular/extracellular water ratio were positively associated with changes in phase angle
Marini et al. (2020) [88]	Assessing the association of classic and BIVA patterns and phase angle with body fluids and fat mass	Cross-sectional study	202 athletes (men: age 21.5 ± 5.0 year; women: age 20.7 ± 5.1 year) engaged in different sports	Foot-to hand at 50 kHz	Dilution techniques (deuterium and bromide) and DXA	(i) Specific BIVA accurately assessed fat mass but no total body water (ii) Classic BIVA accurately assessed total body water but no fat mass (iii) The intracellular/extracellular water ratio were positively associated with phase angle
Campa et al. (2020) [70]	Assessing the ability of BIVA in tracking body fluids changes over the competitive period and vector position in relation to lean soft tissue	Observational study	58 athletes (men: age 18.7 ± 4.0 year; women: age 19.2 ± 6.0 year) engaged in different sports	Foot-to hand at 50 kHz	Dilution techniques (deuterium and bromide) and DXA	(i) Decreases in total body water were accompanied by vector elongations, and vice versa (ii) Lateral vectors lying on the left or right side of the BIVA graph resulted in higher or lower phase angles, indicating more or less soft tissue, respectively (iii) Changes in intracellular/extracellular water ratio were positively associated with changes in phase angle
Francisco et al. (2020) [81]	Assessing the associations of raw bioelectrical parameters with body fluids	Cross-sectional study	202 athletes (men: age 21.5 ± 4.5 year; women: age 20.4 ± 5.2 year) engaged in different sports	Foot-to hand at 50 kHz	Dilution techniques (deuterium and bromide)	(i) Lower R is associated with higher total body water whereas lower Xc is associated with higher extracellular water (ii) The intracellular/extracellular water ratio were positively associated with phase angle

Note: Data are shown as mean ± standard deviation. BIA: bioimpedance analysis; BIVA: bioimpedance vector analysis; DXA: Dual-energy X-ray Absorptiometry; R: resistance; Xc: reactance.

**Table 3:** Predictive equations for estimating body composition in athletes.

Authors	Estimate Variables	Technology and Sampling Frequency	Reference Method	Equation	Note
Matias et al. (2016) [24]	- Total body water - Extracellular water	Foot-to hand at 50 kHz	Dilution techniques (deuterium and bromide)	- Total body water (kg) = 0.286 + 0.195 × stature^2^/R+ 0.385 × body mass + 5.086 × Sex - Extracellular water (kg) = 1.579 + 0.055 × stature^2^/R + 0.127 × body mass + 0.006 × stature^2^/Xc + 0.932 × Sex	where sex is 0 if female or 1 if male, R is resistance, and Xc is reactance
Matias et al. (2020) [25]	Fat-free mass	Foot-to hand at 50 kHz	Four-compartment model	- Fat-free mass (kg) = −2.261 + 0.327 × stature^2^/R + 0.525 × body mass + 5.462 × Sex	where sex is 0 if female or 1 if male, and R is resistance
Sardinha et al. (2020) [26]	- Arms lean soft tissue - Legs lean soft tissue	Foot-to hand at 50 kHz	DXA	- Arms lean soft tissue (kg) = 0.940 × Sex + 0.042 × body mass + 0.080 × stature^2^/R + 0.024 × Xc − 3.927 - Legs lean soft tissue (kg) = 1.983 × Sex + 0.154 × body mass +0.127 × stature^2^/R − 1.147	where sex is 1 if female or 0 if male, R is resistance, and Xc is reactance

Note: DXA: Dual-energy X-ray absorptiometry; R: resistance; Xc: reactance.

**Table 4:** Bioelectrical impedance references for athletes.

Authors	Population	Sample Size	Competitive Period	Technology and Sampling Frequency	R/H	Xc/H	Phase Angle
Micheli et al. (2014) [34]	Male adult elite soccer players	219	first half of the in-season period	Foot-to hand at 50 kHz	252.1 ± 23.1	33.7 ± 3.6	7.7 ± 0.6
Koury et al. (2014) [35]	General male adolescents	195	N/A	Foot-to hand at 50 kHz	302.0 ± 71.0	36.1 ± 6.7	6.9 ± 0.9
Koury et al. (2014) [35]	General adult	90	N/A	Foot-to hand at 50 kHz	252.4 ± 33.8	35.4 ± 4.9	8.0 ± 0.7
Campa and Toselli (2018) [32]	Male adult elite volleyball players	75	Second half of the in-season period	Foot-to hand at 50 kHz	232.1 ± 24.1	31.5 ± 4.3	7.7 ± 0.7
Giorgi et al. (2018) [33]	Male adult elite ciclysts	79	N/A	Foot-to hand at 50 kHz	284.5 ± 31.4	34.9 ± 4.1	7.0 ± 0.7
Campa et al. (2019) [31]	General male adult endurance athletes	165	Off-season period	Foot-to hand at 50 kHz	267.2 ± 28.0	35.5 ± 4.7	7.6 ± 0.8
Campa et al. (2019) [31]	General male adult team sports athletes	576	Off-season period	Foot-to hand at 50 kHz	246.2 ± 32.3	32.9 ± 4.8	7.6 ± 0.8
Campa et al. (2019) [31]	General male velocity/power athletes	375	Off-season period	Foot-to hand at 50 kHz	253.3 ± 32.4	34.2 ± 5.5	7.7 ± 0.8
Campa et al. (2019) [31]	General female adult endurance athletes	76	Off-season period	Foot-to hand at 50 kHz	337.5 ± 42.9	40.1 ± 5.5	6.8 ± 0.8
Campa et al. (2019) [31]	General female adult team sports athletes	187	Off-season period	Foot-to hand at 50 kHz	305.6 ± 37.6	36.3 ± 5.3	6.8 ± 0.8
Campa et al. (2019) [31]	General female velocity/power athletes	177	Off-season period	Foot-to hand at 50 kHz	321.0 ± 46.9	38.0 ± 7.4	7.0 ± 0.8
Toselli et al. (2020) [36]	Youth elite soccer players	178	first part of the preparation period	Foot-to hand at 50 kHz	382.1 ± 81.6	41.3 ± 7.8	6.4 ± 0.8
Bongiovanni et al. (2020) [30]	Male adult elite soccer players	131	End of the preparation period	Foot-to hand at 50 kHz	281.1 ± 20.3	34.6 ± 3.3	8.0 ± 0.5

Note: Data are shown as mean ± standard deviation. R/H: resistance adjusted for height; Xc/H: reactance adjusted for height.

**Figure A1:**
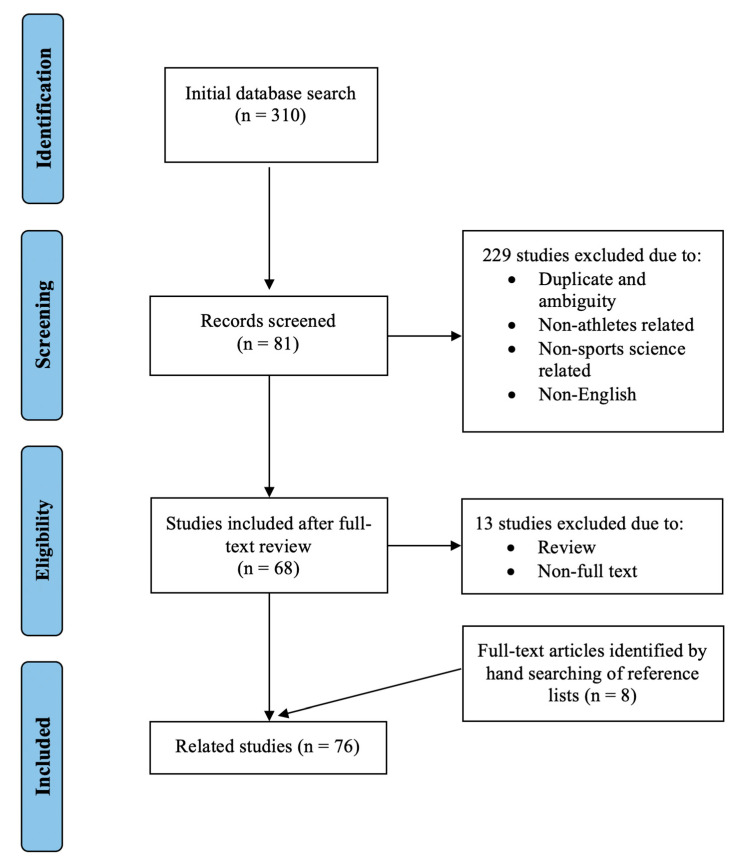
Summary of the search and screening methods implemented in this narrative review.

## Data Availability

No new data were created or analyzed in this study. Data sharing is not applicable to this article.

## Supplementary Materials

The following are available online at https://www.mdpi.com/article/10.3390/nu13051620/s1: Table S1. Complete literature search strategy.

## Appendix A

A systematic search containing terms related to “BIA”, “bioimpedance analysis”, “bioelectric impedance analysis”, “bioelectric impedance vector analysis”, “BIVA”, “bioelectrical phase angle”, and “athletes” was conducted in the following databases: PubMed, SPORT Discus (EBSCO), Medline, Embase, Emcare, Scopus, The Cochrane Library, Web of Science, AUSPORT, and CINHAL (final search 20 December 2020). Articles were required to be peer-reviewed, in full text, and in the English language. Search terms were combined by Boolean logic (AND, OR). Articles were eligible for inclusion if the population contained athletes involved in individual or team sports and if body composition analysis through BIA was performed. No exclusion criteria were based on the participants’ competitive level or age. Articles were excluded if they were reviewed and had duplicate and ambiguous literature. The full search strategy is contained in the Supplementary Materials (Table S1), and an overview of the search and screening process is provided in Figure A1.
