# Supplementary material for: Assessment of Body Composition in Athletes: A Narrative Review of Available Methods with Special Reference to Quantitative and Qualitative Bioimpedance Analysis
Source: Nutrients. 2021 May 12;13(5):1620. doi: 10.3390/nu13051620 (PMC8150618; doi:10.3390/nu13051620)
Supplement: Supplementary file 1 [file nutrients-13-01620-s001.zip › nutrients-1195941-SI.pdf]

Table S1. Complete Search Strategy.

Title of the database searched<br>**PubMed**<br>Name of the database platform<br>**National Center for Biotechnology Information**<br>Complete search strategy<br>**("Athletes"[Mesh] OR "Sports"[Mesh]) OR ("athlete*"[Title/Abstract] OR "player*"[Title/Abstract] AND ("bioimpedance analysis"[Mesh] OR ("BIA*"[Title/Abstract] OR "BIVA*"[Title/Abstract] OR "bioelectrical proprieties*"[Title/Abstract] OR "phase angle*"[Title/Abstract] OR "impedance*"[Title/Abstract] AND ("humans"[MeSH Terms] AND English[lang])**
Title of the database searched<br>**SPORTDiscus**<br>Name of the database platform<br>**EBSCOhost**<br>Complete search strategy<br>**((KW (athletes OR sports) OR SU (athletes OR sports) OR TI (athlete* OR player*) OR AB (athlete* OR player*) AND (KW (bioimpedance analysis OR BIA*) OR SU (bioimpedance analysis) OR TI ("bioimpedance*" OR "BIA*" OR "BIVA*" OR "impedance*" OR "phase angle*" OR "bioelectrical proprieties" OR "resistance" OR "reactance") OR AB ("bioimpedance*" OR "BIA*" OR "BIVA*" OR "impedance*" OR "phase angle*" OR "bioelectrical proprieties" OR "resistance" OR "reactance")))) AND LA english**
Title of the database searched<br>**MEDLINE**<br>Name of the database platform<br>**Ovid**<br>Complete search strategy<br>**(Bioimpedance analysis.sh. or (BIA* or impedance).ti. or (bioimpedance analysis* BIA* or impedance* or bioelectrical proprieties or phase angle or BIVA or bioimpedance vector analysis).ab.) and ((Athletes or Sports).sh. or (athlete* or player* or train* or sport).ab. or (athlete* or player* or train* or sport).ti.) and limit 1 to (english language and humans)**
Title of the database searched<br>**Embase**<br>Name of the database platform<br>**Ovid**<br>Complete search strategy<br>**(Bioimpedance Analysis.sh. or (bioimpedance analysis* or bioimpedance* or BIA* or phase angle or bioelectrical proprieties or BIVA or bioimpedance vector analysis).ti. or (bioimpedance analysis* or BIA* or phase angle or bioelectrical proprieties or BIVA or bioimpedance vector analysis).ab.) and ((Athletes or Sports).sh. or (athlete* or player* or train* or sport).ab. or (athlete* or player* or competit* or train* or sport).ti.) and limit 1 to (english language and humans)**
Title of the database searched<br>**Emcare**<br>Name of the database platform<br>**Ovid**<br>Complete search strategy

**((Bioimpedance Analysis.sh. or (bioimpedance analysis* or BIA* or phase angle or bioelectrical proprieties or BIVA or bioimpedance vector analysis).ti. or (bioimpedance analysis* or BIA* or bioelectrical proprieties or phase angle or BIVA or bioimpedance vector analysis).ab.) and ((Athletes or Sports).sh. or (athlete* or player* or competit* or or train* or sport).ab. or (athlete* or player* or competit* or or train* or sport).ti.) and limit 1 to (english language and humans)**
Title of the database searched<br>**Scopus**<br>Name of the database platform<br>**Elsevier Science Publishers**<br>Complete search strategy<br>**TITLE-ABS-KEY ((("bioimpedance analysis*" OR "BIA *" OR "BIVA" OR "phase angle" OR "bioelectrical proprieties*" OR "bioimpedance vector analysis") AND ("athlete*" OR "player*" OR "competit*" OR "train*" OR "sport"))) AND "human*" AND (LIMIT TO (LANGUAGE , "English"))**
Title of the database searched<br>**The Cochrane Library**<br>Name of the database platform<br>**John Wiley and Sons**<br>Complete search strategy<br>**((("bioimpedance analysis*" OR "BIA*" OR "BIVA" OR "bioimpedance vector analysis" OR "phase angle) AND ("athlete*" OR "player*" OR "competit*" OR "train*" OR "sport*"))) in Title Abstract Keyword - (Word variations have been searched)**
Title of the database searched<br>**Web of Science**<br>Name of the database platform<br>**Clarivate Analytics**<br>Complete search strategy<br>**(TS = ((("bioimpedance analysis*" OR "BIA*" OR "BIVA" OR "bioimpedance vector analysis" OR "phase angle OR "bioelectrical proprieties") AND ("athlete*" OR "player*" OR "competit*" OR "train*" OR "sport*")))) *AND* LANGUAGE: (English)**
Title of the database searched<br>**AUSPORT**<br>Name of the database platform<br>**Informit**<br>Complete search strategy<br>**All Fields, Any Term ("bioimpedance analysis*" OR "BIA*" OR "BIVA" OR "bioimpedance vector analysis" OR "phase angle OR "bioelectrical proprieties") AND ("athlete*" OR "player*" OR "competit*" OR "train*" OR "sport*")**
Title of the database searched<br>**CINAHL**<br>Name of the database platform<br>**EBSCOhost**<br>Complete search strategy<br>**((KW (athletes OR sports) OR SU (athletes OR sports) OR TI (athlete* OR player* OR competit* OR train* OR sport*) OR AB (athlete* OR player* OR competit* OR train* OR sport*)) AND (**

KW (bioimpedance analysis OR BIA*) OR SU (bioimpedance analysis) OR TI (“bioimpedance analysis*” OR “BIA *” OR “BIVA OR “bioimpedance vector analysis” OR “phase angle” OR “bioelectrical proprieties”) OR AB (“bioimpedance analysis*” OR “BIA*” OR “BIVA” OR “bioimpedance vector analysis” OR “phase angle” OR “bioelectrical proprieties”))) AND LA english AND DH Human
